# Targeting Thymidylate Synthase and tRNA-Derived Non-Coding RNAs Improves Therapeutic Sensitivity in Colorectal Cancer

**DOI:** 10.3390/antiox11112158

**Published:** 2022-10-31

**Authors:** Changwon Yang, Jisoo Song, Sunwoo Park, Jiyeon Ham, Wonhyoung Park, Hahyun Park, Garam An, Taeyeon Hong, Hee Seung Kim, Gwonhwa Song, Whasun Lim

**Affiliations:** 1 Department of Biotechnology, Institute of Animal Molecular Biotechnology, College of Life Sciences and Biotechnology, Korea University, Seoul 02841, Korea; 2 Department of Biological Sciences, College of Science, Sungkyunkwan University, Suwon 16419, Korea; 3 Department of Plant & Biomaterials Science, Gyeongsang National University, Jinju 52725, Korea; 4 Department of Obstetrics and Gynecology, College of Medicine, Seoul National University, Seoul 03080, Korea

**Keywords:** colorectal cancer, quercetin, thymidylate synthase, p53, 5-fluorouracil

## Abstract

Some colorectal cancer (CRC) patients are resistant to 5-fluorouracil (5-FU), and high expression levels of thymidylate synthase (TS) contribute to this resistance. This study investigated whether quercetin, a representative polyphenol compound, could enhance the effect of 5-FU in CRC cells. Quercetin suppressed TS levels that were increased by 5-FU in CRC cells and promoted the expression of p53. Quercetin also induced intracellular and mitochondrial reactive oxygen species (ROS) production and Ca^2+^ dysregulation in a 5-FU-independent pathway in CRC cells. Furthermore, quercetin decreased mitochondrial membrane potential in CRC cells and inhibited mitochondrial respiration. Moreover, quercetin regulated the expression of specific tiRNAs, including tiRNA^HisGTG^, and transfection of a tiRNA^HisGTG^ mimic further enhanced the apoptotic effect of quercetin in CRC cells. An enhanced sensitivity to 5-FU was also confirmed in colitis-associated CRC mice treated with quercetin. The treatment of quercetin decreased survival rates of the CRC mouse model, with reductions in the number of tumors and in the disease activity index. Also, quercetin suppressed TS and PCNA protein expression in the distal colon tissue of CRC mice. These results suggest that quercetin has the potential to be used as an adjuvant with 5-FU for the treatment of CRC.

## 1. Introduction

The most important chemotherapeutic agent for the treatment of CRC is 5-fluorouracil (5-FU), a synthetic fluorinated pyrimidine analog. DNA replication is suppressed by 5-FU through the inhibition of thymidylate synthase (TS) activity [1]. CRC cells that are resistant to 5-FU express high levels of TS, which may contribute to their resistance [2,3]. Therefore, identifying compounds that target TS and elucidating their mechanism of action could help enhance the sensitivity of CRC cells to 5-FU [4].

The potential of quercetin as a therapeutic strategy in CRC is widely recognized. Quercetin inhibits cell proliferation and induces apoptosis by regulating various signaling pathways in CRC cells and may alleviate the inflammatory response [5,6]. However, the detailed mechanism by which quercetin enhances sensitivity to 5-FU remains unknown. A previous study verified that quercetin potentiates 5-FU-induced apoptosis in CRC cells according to p53 status [7]. At the same time, TS can inhibit p53 at the translational level, and in a study, we suggested that a compound caused mitochondrial-mediated apoptosis in CRC cells by targeting the TS-p53 axis [8,9]. Therefore, this study investigated whether quercetin regulates the expression levels of TS, p53, and proteins involved in the cell cycle and in proliferation, in addition to analyzing mitochondrial dysfunction in CRC cells.

tRNA-derived non-coding RNAs play an important role in the development of various cancers [10]. In particular, tiRNAs are specifically cleaved by angiogenin from the anticodon loop of tRNA in response to external stress. A sequencing analysis performed on CRC tissues revealed that the expression levels of various tiRNAs were specifically increased or decreased [11]. A recent study reported that tiRNA^ProTGG^ is associated with poor survival in CRC patients [12]. Additionally, the expression of specific tRNA-derived fragments in CRC cells may be regulated by the action of Dicer1 under the hypoxic conditions thought to be associated with cell invasiveness and epithelial-to-mesenchymal transition [13,14]. However, it is unclear whether specific tiRNAs affect the viability and apoptosis of CRC cells. Therefore, in this study, tiRNAs regulated by quercetin were identified and mimics were transfected into CRC cells to determine their effect on apoptosis.

The purpose of this study was to elucidate whether quercetin enhances the effect of 5-FU on cell viability, cell proliferation, and apoptosis in CRC cells, with a focus on TS and p53 protein. Moreover, this study investigated whether quercetin is involved in mitochondrial-mediated apoptosis, oxidative stress, and Ca^2+^ dysregulation in CRC cells. We also verified the effect of quercetin on the expression levels of candidate tiRNAs and the function of tiRNAs in CRC cells. In addition, we investigated the tumor growth inhibitory effect of quercetin in colitis-associated CRC mice treated with azoxymethane (AOM) and dextran sodium sulfate (DSS).

## 2. Materials and Methods

### 2.1. Cell Culture

Human CRC-derived cell lines, HCT116 and HT29, and a normal colon fibroblast line, CCD-18Co, were purchased from the Korean Cell Line Bank (Seoul, Republic of Korea). To establish a 5-FU-resistant HCT116 (HCT116-5-FUR) cell line, parental HCT116 cells were first treated with 0.25 μM 5-FU, and the concentration was doubled every three passages. Dead cells were removed from each subculture, and cells surviving 5-FU treatment were cultured for at least 6 months.

### 2.2. Measurement of Cell Viability and Proliferation

An MTT Cell Proliferation Kit (Roche, Basel, Switzerland) and ELISA kit for BrdU (Roche) were used to measure cell viability and cell proliferation, respectively, as previously described [15,16]. For the proliferation assay, 10 μM bromo-2′-deoxyuridine (BrdU) was added to the cell culture, and the cells were incubated for an additional 2 h at 37 °C. Subsequently, the cells were fixed, incubated with anti-BrdU peroxidase, and detected using 3,3′,5,5′-tetramethylbenzidine substrate, according to the manufacturer’s instructions. For the cell viability assay, cells were incubated with MTT tetrazolium salt at 37 °C for 4 h, followed by incubation in solubilization buffer at 37 °C overnight. Absorbance was measured at 560 and 650 nm using a microplate reader.

### 2.3. Annexin V and Propidium Iodide (PI) Staining

An annexin V apoptosis detection kit I (BD Biosciences, Franklin Lakes, NJ, USA) was used to analyze changes in apoptosis in CRC cells, as previously described [15]. The cells were then incubated with FITC annexin V and PI for 15 min at room temperature. In addition, PI staining with RNase A treatment was performed to investigate cell cycle progression in CRC cells.

### 2.4. Cell Migration Assay

Cell migration was analyzed using a culture-insert 2 well in µ-dish (Ibidi GmbH, Gräfelfing, Germany). After CRC cells in the wells were treated with quercetin, the wells were removed and cell migration was observed under a microscope. Cell migration was quantified based on the distance between cell populations on both sides.

### 2.5. Cell Growth Observation

To analyze cell growth in a 3D environment, a spheroid culture system was established using the hanging drop method. Cells diluted in growth medium to a concentration of 1 × 10^5^/mL were dropped onto the lid of a culture dish with 5-FU and quercetin. A Petri dish was filled with PBS to serve as a hydration chamber. After 3 days, cell growth and shape were analyzed under a microscope. Colony number and average colony area were quantified via ImageJ software. In addition, the growth patterns of cells growing in Matrigel in main wells were analyzed using 96-well plates with satellite wells. Quercetin and 5-FU were placed in satellite wells for 48 h to penetrate the Matrigel and expose cells.

### 2.6. Protein Expression Analysis

Western blotting was performed to analyze the expression levels of proteins in CRC cells, as previously described [15]. α-Tubulin (TUBA) expression was used to normalize the data and Image Lab software (Bio-Rad, Hercules, CA, USA) was used for quantification. Immunofluorescence was used to visualize the expression levels of TS protein in CRC cells, as previously described [17]. Based on the fluorescence images obtained through confocal microscopy, the expression levels of TS were quantified by dividing its fluorescence intensity by the fluorescence intensity of DAPI using MetaMorph software (Molecular Devices, San Jose, CA, USA).

### 2.7. Analysis of Mitochondrial Dysfunction

Harvested cells were stained with JC-1 dye at 37 °C for 20 min. Thereafter, the relative loss of mitochondrial membrane potential (MMP) was analyzed based on the fluorescence intensity measured by a FACSCalibur™ (BD Biosciences) flow cytometer. To analyze intracellular metabolic flux, the XF24 extracellular flux assay kit (Agilent Technologies, Santa Clara, CA, USA) was used. Basal respiration, ATP production, and maximal respiration, as determined by the administration of each respective solution, were analyzed to quantify mitochondrial stress. Metabolic potential was analyzed by measuring the energy phenotype of cells that met their energy demands via respiration and glycolysis.

### 2.8. Intracellular ROS and Ca^2+^ Detection

To measure intracellular ROS production, we used 2′,7′-dichlorofluorescin diacetate solution, as previously described [15]. To measure mitochondrial-specific ROS generation in detail, the MitoSOX red mitochondrial superoxide indicator (Invitrogen, Waltham, MA, USA), which specifically targets mitochondria, was used to stain the cells at 37 °C for 10 min. In addition, to specifically analyze the generation of intracellular superoxide, dihydroethidium (DHE) was used to stain the cells and analyzed by FACSCalibur™. Intracellular and mitochondrial Ca^2+^ levels were analyzed using Fluo-4 and Rhod-2 dyes, respectively, as previously described [15].

### 2.9. tiRNA Detection

MINTbase (https://cm.jefferson.edu/MINTbase/, accessed on 10 October 2022) software was used to obtain the sequences of candidate tiRNAs and mature tRNA sources and calculate reads per million (RPM) based on tiRNA type and anticodon [18]. RNA extracted from the colon tissues of CRC mice were synthesized into cDNA using the polyadenylation method. To confirm the expression of five candidate tiRNAs, quantitative PCR was performed using miRNA QPCR Master Mix (Agilent Technologies, Santa Clara, CA, USA), the forward primer for each tiRNA, and the universal primer included in the cDNA synthesis kit. The expression of U6 snRNA was used to normalize the data, and the 2^−ΔΔCT^ method was used for the relative quantification of tiRNA expression levels. To obtain images of the tiRNA products, we used the digoxygenin (DIG)-labeled DNA probe-based small RNA detection method described in our previous study [19].

### 2.10. AOM/DSS CRC Model

The previously described method of AOM and DSS administration was modified to establish a mouse model of inflammatory CRC [20]. All animal handling procedures were conducted according to the IACUC guidelines (KMU-2021-02). Male 6-week-old C57BL/6 mice were purchased from DBL (Eumseong, Republic of Korea) and intraperitoneally injected with 0.1 mL of AOM solution diluted in saline to a concentration of 10 mg/kg on day 8, after an adaptation period of 1 week. Normal drinking water was then provided for 1 week, after which 2.5% DSS solution was provided for 5 days. Mice then received normal drinking water for 2 weeks. Mice were sacrificed following three cycles of 1 week of DSS solution and 2 weeks of normal drinking water. Mice were then divided into five groups, with 24 mice in each group. Group 1 was a normal control group, intraperitoneally injected with saline solution instead of AOM/DSS. Group 2 was an AOM/DSS CRC model, which served as a control group without 5-FU and quercetin administration. Group 3 included AOM/DSS CRC mice intraperitoneally injected with 5-FU at a concentration of 20 mg/kg once every 3 days, starting from day 8. Group 4 included AOM/DSS CRC mice administered with 30 mg/kg quercetin orally, starting daily from day 8. In Group 5, AOM/DSS CRC model mice were intraperitoneally injected with 20 mg/kg 5-FU every 3 days from day 8 and administered 30 mg/kg quercetin orally every day. The same dose of saline was applied as a control for 5-FU and quercetin administration. Survival rates of CRC mice were recorded during the experiment, and body weights were measured once every 3 days. In addition, rectal bleeding and stool consistency in mice were observed on the last day of each cycle as evidence of colonic damage. The disease activity index (DAI) for colonic damage was scored according to body weight loss, rectal bleeding, and stool consistency, as previously described [21].

### 2.11. Histological Analysis

To visualize the nucleus and cytoplasm on deparaffinized tissue slides, distal colon tissues were stained with hematoxylin and eosin (H&E). In addition, acidic mucin in goblet cells was visualized by staining with 1% Alcian blue at pH 2.5. The tissue slides were then dehydrated in serial dilutions of alcohol and mounted for microscopy using a mounting medium. Histological scoring analysis was performed based on intestinal damage, as follows: 1 point for loss of goblet cells, 2 points for loss of goblet cells in large areas, 3 points for loss of crypts, and 4 points for loss of crypts in large areas. The immunocytochemical localization of TS and proliferating cell nuclear antigen (PCNA) in the distal colon was detected using an avidin-biotin complex (ABC) kit (Vector Laboratories, Newark, CA, USA).

### 2.12. Quantitative PCR

Quantitative PCR was performed to quantify mRNA expression levels, as previously described [22]. RNA was extracted from the distal colon of CRC mice using Trizol reagent and synthesized into cDNA using AccuPower RT Pre-Mix (Bioneer, Daejon, Republic of Korea). Gene expression was determined using SYBR^®^ Green (Sigma, St. Louis, MO, USA) and a StepOnePlus^™^ Real-Time PCR System (Applied Biosystems, Waltham, MA, USA).

### 2.13. Immune Cell Analysis

Spleens from CRC mice were collected and immersed in RPMI medium containing 5% FBS and crushed in a 70 μm cell strainer for immune cell analysis. The crushed spleen and fluid were centrifuged at 300× *g* for 5 min. The pellet was diluted in RPMI medium containing 5% FBS for cell counting. Antibodies against CD45, Gr-1, and CD11b were used to count myeloid-derived suppressor cells (MDSCs), and antibodies against CD45, CD4, CD25, and FOXP3 were used to count Tregs; all antibodies were incubated with cells on ice for 20 min. For cells resuspended in PBS, the fluorescence signal of each antibody was measured using FACSVerse™ (BD Biosciences).

### 2.14. Statistics

Statistical analyses were performed using analysis of variance according to the general linear model with SAS statistical software. All experiments were repeated at least three times. A *p* value of <0.05 (two-tailed) was considered statistically significant for all tests.

## 3. Results

### 3.1. Quercetin Potentiates the Effects of 5-FU on Characteristics of CRC Cells

**Figure 1:**
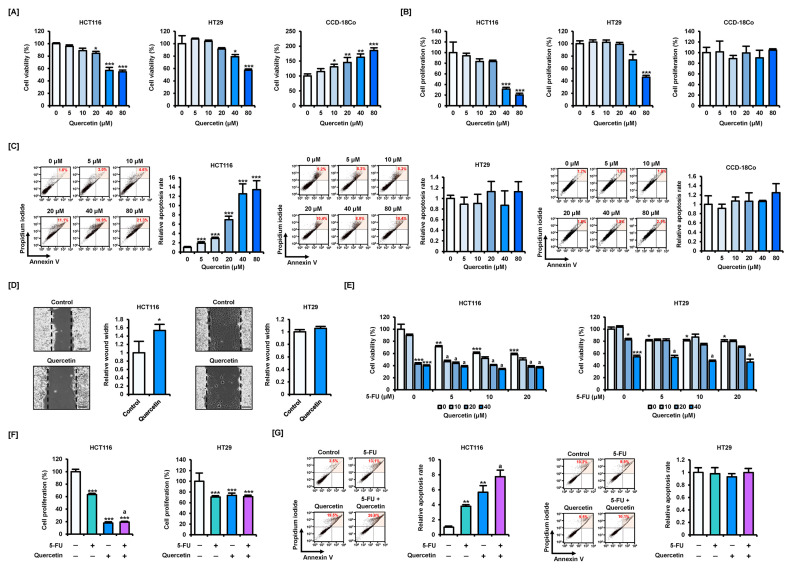
Effects of quercetin on the viability and proliferation of colorectal cancer (CRC) cells. (**A**) Dose-dependent effects of quercetin (0, 5, 10, 20, 40, and 80 μM) on the viability of HCT116, HT29, and CCD18-Co cells over 48 h. (**B**) Dose-dependent effects of quercetin (0, 5, 10, 20, 40, and 80 μM) on the proliferation of HCT116, HT29, and CCD18-Co cells over 48 h. (**C**) Dose-dependent effects of quercetin (0, 5, 10, 20, 40, and 80 μM) on apoptosis of HCT116, HT29, and CCD18-Co cells over 48 h. Annexin V and propidium iodide (PI) fluorescence values were estimated by flow cytometry. The percentage of cells corresponding to the upper right of the quadrant was used to compare the relative number of apoptotic cells. (**D**) Effects of quercetin (40 μM) on the migration of HCT116 and HT29 cells over 12 h. Scale bar represents 100 μm. (**E**) Effects of dose-dependent combination treatment of 5-fluorouracil (5-FU, 0, 5, 10, and 20 μM) and quercetin (0, 10, 20, and 40 μM) on viability of HCT116 and HT29 cells over 48 h. (**F**) Effects of 5-FU (20 μM) and quercetin (40 μM) combination treatment on proliferation of HCT116 and HT29 cells over 48 h. (**G**) Effects of 5-FU (20 μM) and quercetin (40 μM) combination treatment on apoptosis of HCT116 and HT29 cells over 48 h. Annexin V and PI fluorescence values were estimated by flow cytometry. The percentage of cells corresponding to the upper right of the quadrant was used to compare the relative number of apoptotic cells. Data are presented as representatives of the results of three independent experiments. Asterisk indicates a statistically significant difference compared with untreated control cells (*** *p* < 0.001; ** *p* < 0.01; * *p* < 0.05). The symbol ‘a’ indicates a significant effect of the combination treatment compared with the treatment with 5-FU alone (*p* < 0.05).

We first analyzed the effects of quercetin on cell viability, proliferation, and apoptosis in CRC cells and normal colon cells. Quercetin inhibited the viability of CRC cells in a dose-dependent manner (Figure 1A). Treatment with quercetin at concentrations higher than 20 μM significantly affected the viability of HCT116 cells and concentrations higher than 40 μM significantly affected the viability of HT29 cells. In HCT116 cells and HT29 cells, 40 μM of quercetin reduced cell viability by 43.2 and 21.0%, respectively. The IC_50_ values of quercetin for the viability of HCT16 and HT29 cells were 24.6 and 49.6 μM, respectively. Interestingly, cell viability increased in a dose-dependent manner in response to quercetin treatment in the normal colon cell line, CCD-18Co. Quercetin also reduced the proliferation of CRC cells, whereas it did not affect the proliferation of CCD-18Co cells (Figure 1B). Treatment with 40 μM of quercetin inhibited cell proliferation by 68.6 and 26.2% in HCT116 and HT29 cells, respectively. The IC_50_ values of quercetin for the proliferation of HCT16 and HT29 cells were 28.8 and 40.3 μM, respectively. Based on the results of the reduction in the viability and proliferation of CRC cells following the dose-dependent treatment of quercetin, we set the optimal concentration for a single treatment of quercetin to be 40 μM. Next, we analyzed the effect of quercetin on apoptosis by staining cells with annexin V and PI (Figure 1C). In HCT116 cells, quercetin increased the proportion of apoptotic cells in a dose-dependent manner. The apoptosis rate increased by 12.5-fold in cells treated with 40 μM of quercetin compared with that of the control group. Interestingly, quercetin did not have a significant effect on apoptosis in either HT29 or CCD-18Co cells. Quercetin also increased the wound width in HCT116 cells, suggesting that it inhibited cell migration (Figure 1D). However, quercetin did not significantly affect migration of HT29 cells. These results suggest that although quercetin reduces the viability of CRC cells, their effect on apoptosis and migration differs depending on cell type.

Next, we analyzed changes in cell viability following dose-dependent treatment with 5-FU and quercetin (Figure 1E). As expected, both 5-FU and quercetin alone significantly inhibited the viability of CRC cells, with a stronger effect in HCT116 cells. In addition, quercetin further potentiated the reduction in the viability of CRC cells when co-treated with 5-FU. In particular, the treatment of HT29 cells with 40 μM of quercetin significantly enhanced the effect of 5-FU on cell viability. Based on these results, we set optimal treatment concentrations of 5-FU and quercetin to 20 and 40 μM, respectively. In HCT116 cells, compared with 5-FU treatment alone, additional treatment with quercetin significantly inhibited cell proliferation (Figure 1F). In HT29 cells, a synergistic effect on cell proliferation was not observed following combined treatment with 5-FU and quercetin; however, both 5-FU and quercetin alone or in combination significantly inhibited cell proliferation compared with the control group. Meanwhile, quercetin further potentiated apoptosis induced by 5-FU in HCT116 cells (Figure 1G). However, in HT29 cells, treatment with 5-FU and quercetin alone or in combination had no effect on apoptosis, suggesting that this effect is dependent on the characteristics of CRC cells. These results suggest that quercetin can enhance the effects of 5-FU on CRC cells. However, there is a marked difference in its effect on apoptosis depending on cell type, suggesting that a more detailed analysis of the anticancer mechanism of quercetin is needed.

### 3.2. Quercetin Overcomes 5-FU Resistance by Regulating the Expression of TS and p53 in CRC Cells

**Figure 2:**
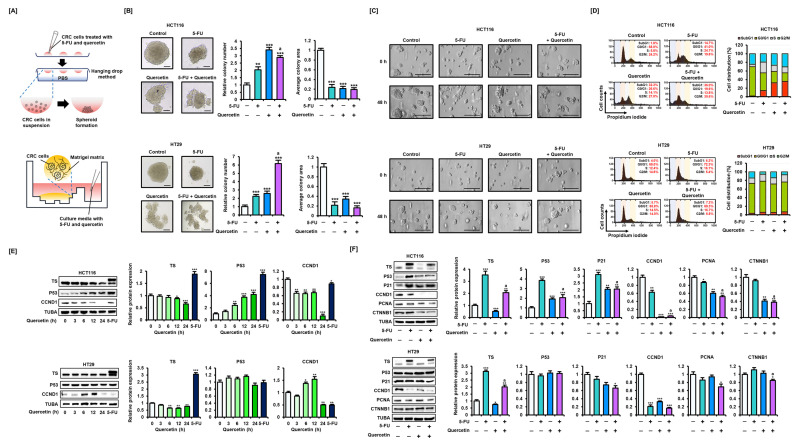
Effects of quercetin on alleviating the expression of target proteins in CRC cells. (**A**) A schematic diagram of spheroid formation through hanging drop method (top) and 3D tumor environment establishment in Matrigel following treatment on satellite wells (bottom). (**B**) Microscopic observation of tumor growth inhibition in spherical cancer cell colonies following the combined treatment of 5-FU (20 μM) and quercetin (40 μM) for 48 h. Scale bar represents 100 μm. ImageJ software was used for a relative comparison of colony number and area. (**C**) Effects of 5-FU (20 μM) and quercetin (40 μM) in satellite wells on cancer cells growing in Matrigel in main wells. Scale bar represents 100 μm. (**D**) Cell cycle distribution following combined treatment of 5-FU (20 μM) and quercetin (40 μM), determined by PI staining and expressed as the proportions of cells in sub-G1, G1, S, and G2/M phases over 48 h. (**E**) Expression levels of TS, p53, and CCND1 in HCT116 and HT29 cells following time-dependent treatment of quercetin (0, 3, 6, 12, and 24 h) at a concentration of 40 μM, as analyzed by Western blot. Protein expression levels following 5-FU (20 μM) treatment alone are also presented. (**F**) Expression of TS, p53, P21, CCND1, PCNA, and CTNNB1 in HCT116 and HT29 cells following combined treatment of 5-FU (20 μM) and quercetin (40 μM), as analyzed by Western blot over 24 h. Expression of TUBA was used to normalize the data. Data are presented as representatives of the results of three independent experiments. Asterisk indicates a statistically significant difference compared with untreated control cells (*** *p* < 0.001; ** *p* < 0.01; * *p* < 0.05). The symbol ‘a’ indicates a significant effect of combination treatment compared with the treatment with 5-FU alone (*p* < 0.05).

Next, we established a spheroid culture and a 3D tumor environment using Matrigel, following treatment in satellite wells, to observe tumor growth patterns in response to 5-FU and quercetin (Figure 2A). Exposure to 5-FU and quercetin during colony formation inhibited tumor growth in CRC cells (Figure 2B). The addition of quercetin to 5-FU treatment significantly inhibited the formation of large-volume colonies. We also confirmed through microscopic observation that the administration of 5-FU and quercetin via satellite wells inhibited the growth of HCT116 cells in Matrigel in the main well, although the effect was insignificant in HT29 cells (Figure 2C). Next, the effects of 5-FU and quercetin on the cell cycle in CRC cells were analyzed (Figure 2D). In HCT116 cells, treatment with 5-FU and quercetin alone or in combination increased the proportion of cells in the sub-G1 phase, suggesting the initiation of the apoptotic pathway. Moreover, quercetin caused G2/M phase arrest in HCT116 cells but did not affect the cell cycle in HT29 cells. These results suggest that quercetin inhibited the growth of CRC cells by regulating the cell cycle, but was dependent on cell type.

We analyzed the time-dependent expression levels of TS (which is involved in 5-FU resistance) and p53 and CCND1 (which are involved in apoptosis and cell cycle regulation), following treatment with quercetin (Figure 2E). We found that quercetin decreased the intensity of the lower band, which is thought to indicate the expression level of unbounded TS, in a time-dependent manner in CRC cells. Meanwhile, quercetin increased the expression level of p53 in HCT116 cells, but did not affect the expression level of p53 in HT29 cells harboring the p53 mutation, as with 5-FU treatment. In addition, quercetin treatment for 24 h decreased the expression levels of CCND1 in CRC cells. Next, we investigated the expression levels of target proteins after co-treating CRC cells with quercetin and 5-FU (Figure 2F). 

**Figure 3:**
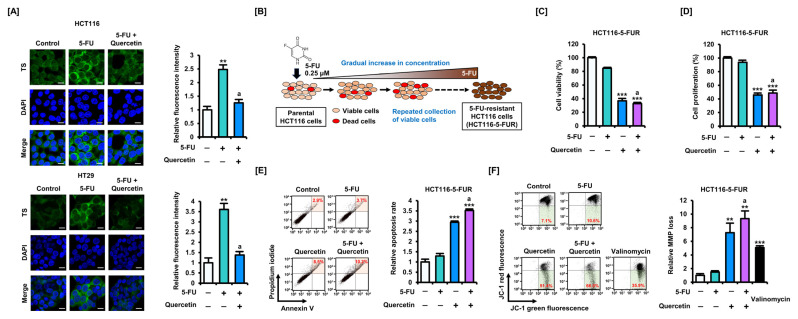
Effect of quercetin on alleviating 5-FU resistance in CRC cells. (**A**) Regulation of TS expression by treatment with 5-FU (20 μM) and quercetin (40 μM), as analyzed by immunofluorescence (green) over 24 h. Nuclei were counterstained with DAPI (blue). The green fluorescence value measured with MetaMorph software was divided by the blue fluorescence value to quantify fluorescence intensity. The scale bars represent 40 μm. (**B**) Schematic diagram of the establishment of HCT116 cells resistant to 5-FU (HCT116-5-FUR). (**C**) Effects of 5-FU (20 μM) and quercetin (40 μM) combination treatment on cell viability in HCT116-5-FUR cells over 48 h. (**D**) Effects of 5-FU (20 μM) and quercetin (40 μM) combination treatment on proliferation of HCT116-5-FUR cells over 48 h. (**E**) Effects of 5-FU (20 μM) and quercetin (40 μM) combination treatment on apoptosis of HCT116-5-FUR cells over 48 h. Annexin V and PI fluorescence values were estimated by flow cytometry. The percentage of cells corresponding to the upper right of the quadrant was used to compare the relative number of apoptotic cells. (**F**) Effects of 5-FU (20 μM) and quercetin (40 μM) combination treatment on mitochondrial membrane potential (MMP) loss in HCT116-5-FUR cells over 48 h. JC-1 fluorescence values were estimated by flow cytometry. The percentage of cells corresponding to the lower right was used for relative comparisons. Asterisk indicates a statistically significant difference compared with untreated control cells (*** *p* < 0.001; ** *p* < 0.01). The symbol ‘a’ indicates a significant effect of combination treatment compared with treatment with 5-FU alone (*p* < 0.05).

As previously reported, 5-FU increased expression levels of both bound and unbound TS in HCT116 and HT29 cells [9]. Co-treatment with 5-FU and quercetin significantly attenuated the increased expression of TS induced by 5-FU. Treatment with 5-FU or quercetin alone increased the expression levels of p53 and its downstream protein, p21, in HCT116 cells. In CRC cells, quercetin significantly reduced the expression levels of CCND1, and in HCT116 cells specifically, CCND1 expression levels were significantly reduced with the combination treatment compared with those treated with 5-FU alone. Compared with 5-FU treatment alone, co-treatment with quercetin also significantly suppressed PCNA expression in CRC cells. Moreover, compared with the 5-FU treatment alone, the expression of β-catenin (CTNNB1), a representative protein that induces CRC progression, also decreased in response to the combined treatment with 5-FU and quercetin. We performed immunofluorescence to closely observe changes in the expression levels of TS in CRC cells (Figure 3A). As in the Western blot analysis, 5-FU increased the fluorescence intensity of TS, whereas co-treatment with quercetin lowered the intensity to control levels.

As our previous study verified that high expression levels of TS could induce resistance to 5-FU, we generated HCT116-5-FUR cells by treating HCT116 cells with progressively increasing concentrations of 5-FU [9] (Figure 3B). Compared with that of parental HCT116 cells, the viability of HCT116-5-FUR cells was reduced to a lower extent following 5-FU treatment (Figure S1). However, treatment with quercetin reduced the viability of HCT116-5-FUR cells to a level comparable with that of parental HCT116 cells (Figure 3C). Moreover, although an effect on cell proliferation was barely detected in HCT116-5-FUR cells treated with 5-FU, proliferation was significantly inhibited in these cells in response to quercetin treatment (Figure 3D). The apoptosis assay also suggested that co-treatment with quercetin could overcome 5-FU resistance in CRC cells (Figure 3E). As mitochondria may be involved in drug resistance, we analyzed whether quercetin could modulate the MMP levels that were largely unchanged by 5-FU in HCT116-5-FUR cells (Figure 3F). We found that quercetin induced a significant loss in MMP when treated alone or in combination with 5-FU. These results suggest that quercetin can modulate the expression of TS and thereby alleviate 5-FU resistance by inducing mitochondrial-mediated apoptosis.

### 3.3. Quercetin Induces Mitochondrial Dysfunction in CRC Cells and Enhances Sensitivity to 5-FU

As the previous results suggested that quercetin may improve the response of 5-FU-resistant CRC cells through a mitochondrial-targeting mechanism, we analyzed whether quercetin regulated mitochondrial function.

**Figure 4:**
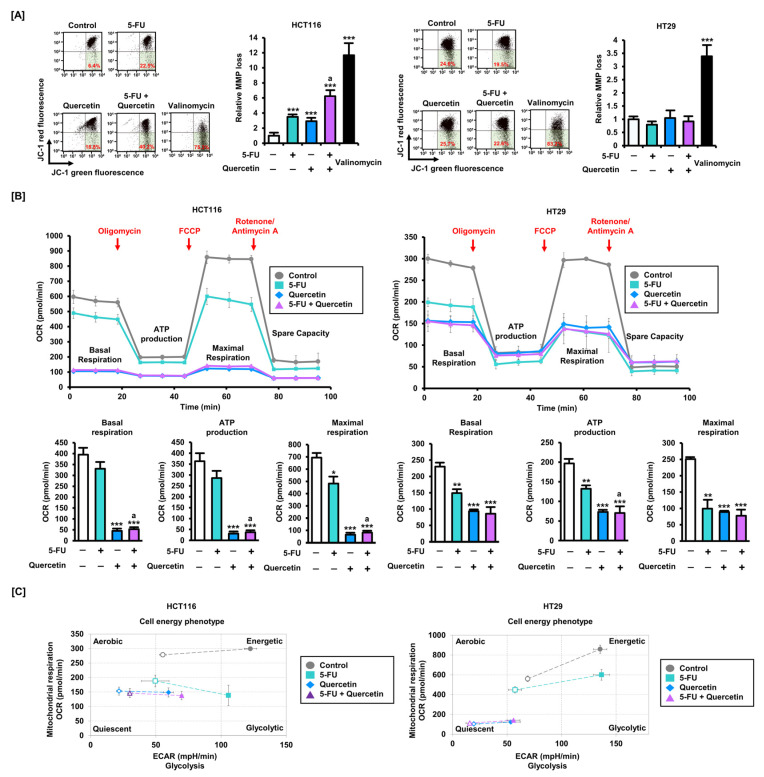
Quercetin induces mitochondrial dysfunction in CRC cells. (**A**) Effects of 5-FU (20 μM) and quercetin (40 μM) combination treatment on mitochondrial membrane potential (MMP) loss in HCT116 and HT29 cells over 48 h. JC-1 fluorescence values were estimated by flow cytometry. The percentage of cells corresponding to the lower right was used for relative comparisons. (**B**) Mitochondrial respiration index following the combined treatment of 5-FU (20 μM) and quercetin (40 μM) was analyzed by Seahorse assay. (**C**) Changes in cellular energy expression following the combined treatment of 5-FU (20 μM) and quercetin (40 μM) were indicated. Data are presented as representatives of the results of three independent experiments. Asterisks indicate statistically significant differences compared with untreated controls (*** *p* < 0.001; ** *p* < 0.01; * *p* < 0.05). The symbol ‘a’ indicates a significant difference between combination treatment and 5-FU treatment alone (*p* < 0.05).

First, we found that quercetin significantly increased the loss of MMP in HCT116 cells when treated in combination with 5-FU or alone (Figure 4A). Interestingly, similar to the apoptosis assay, neither 5-FU nor quercetin had any effect on MMP in HT29 cells. Meanwhile, we performed a Seahorse Mito Stress assay to investigate the effect of quercetin on mitochondrial function in CRC cells (Figure 4B). Oligomycin inhibits ATP synthase, leading to a decrease in OCR. FCCP disrupts the protein gradient, disturbs MMP, and leads to maximum oxygen consumption. A mixture of rotenone and antimycin A shuts down mitochondrial respiration. At each stage, basal respiration, ATP production, and maximal respiration, quantified by OCR levels, were all significantly reduced in both HCT116 and HT29 cells by quercetin treatment. It is well-known that glycolysis is a major metabolic mechanism for energy production and anabolic growth in cancer cells. Quercetin inhibited mitochondrial respiration, represented by OCR, and glycolysis rates, represented by ECAR, in HCT116 and HT29 cells (Figure 4C). These results suggest that quercetin may enhance the effects of 5-FU by inducing mitochondrial dysfunction in CRC cells.

### 3.4. Quercetin Induces ROS Production and Abnormal Regulation of Ca^2+^ in CRC Cells

**Figure 5:**
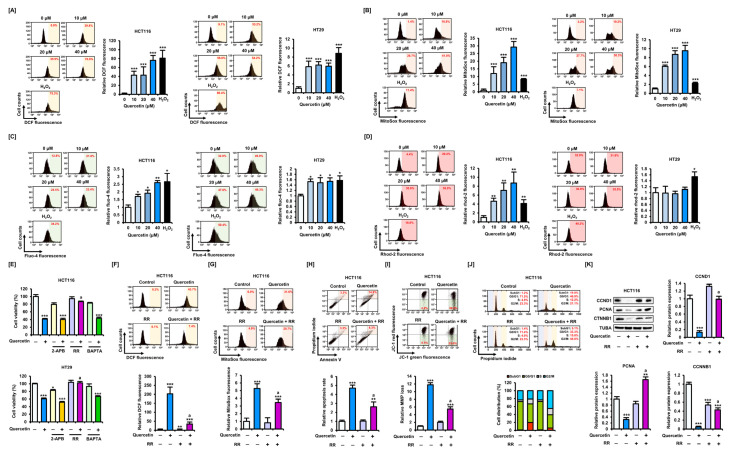
Quercetin causes oxidative stress and an increase in Ca^2+^ in CRC cells. (**A**) Effects of quercetin on ROS production in HCT116 and HT29 cells over 1 h. Flow cytometry was used to estimate 2′,7′-dichlorofluorescein (DCF) fluorescence values. (**B**) Effects of quercetin on mitochondrial ROS production in HCT116 and HT29 cells over 1 h. MitoSOX fluorescence values were estimated by flow cytometry. (**C**) Effects of quercetin on intracellular Ca^2+^ levels in HCT116 and HT29 cells over 1 h. Fluo-4 fluorescence values were estimated by flow cytometry. (**D**) Effects of quercetin on mitochondrial Ca^2+^ levels in HCT116 and HT29 cells over 1 h. Rhod-2 fluorescence values were estimated by flow cytometry. (**E**) Analysis of changes in cell viability after combined treatment of 2-APB, RR, and BAPTA with quercetin (40 μM) over 48 h in HCT116 and HT29 cells. (**F**) Effects of quercetin (40 μM) and RR (16 μM) combination treatment on intracellular ROS production in HCT116 cells over 1 h. DCF fluorescence values were estimated by flow cytometry. (**G**) Effects of quercetin (40 μM) and RR (16 μM) combination treatment on mitochondrial ROS production in HCT116 cells over 1 h. MitoSox fluorescence values were estimated by flow cytometry. (**H**) Effects of quercetin (40 μM) and RR (16 μM) combination treatment on apoptosis in HCT116 cells over 48 h. Annexin V and PI fluorescence values were estimated by flow cytometry. The percentage of cells corresponding to the upper right of the quadrant was used to compare the relative number of apoptotic cells. (**I**) Effects of quercetin (40 μM) and RR (16 μM) combination treatment on mitochondrial membrane potential (MMP) loss in HCT116 cells over 48 h. JC-1 fluorescence values were estimated by flow cytometry. The percentage of cells corresponding to the lower right was used for relative comparisons. (**J**) Cell cycle distribution following combined treatment of quercetin (40 μM) and RR (16 μM) was determined by PI staining and expressed as proportions of cells in sub-G1, G1, S, and G2/M phases over 48 h. (**K**) Expression of CCND1, PCNA, and CTNNB1 in HCT116 cells following combined treatment of quercetin (40 μM) and RR (16 μM), as analyzed by Western blot over 24 h. Expression of TUBA was used for normalization. Asterisks indicate statistically significant differences compared with untreated controls (*** *p* < 0.001; ** *p* < 0.01; * *p* < 0.05). The symbol ‘a’ indicates a significant difference between combination treatment and quercetin treatment alone (*p* < 0.05).

Oxidative stress can induce mitochondrial dysfunction, a useful therapeutic strategy in cancer cells. Therefore, we analyzed whether quercetin could induce oxidative stress in CRC cells. First, we found that quercetin induced a significant increase in intracellular ROS production in CRC cells (Figure 5A). Specifically, an increase in ROS production was induced in response to quercetin in the mitochondria of HCT116 and HT29 cells (Figure 5B). Moreover, quercetin significantly increased superoxide production at concentrations of 20 μM or higher (Figure S2). Oxidative stress in cancer cells can lead to mitochondrial dysfunction through excessive Ca^2+^ influx into the mitochondria. Therefore, we investigated whether quercetin could modulate Ca^2+^ levels in CRC cells. Quercetin induced significant increases in intracellular Ca^2+^ in HCT116 and HT29 cells, and significantly increased mitochondrial Ca^2+^ levels in HCT116 cells, but not in HT29 cells (Figure 5C,D). These results are consistent with the finding that quercetin had little effect on MMP loss in HT29 cells. Furthermore, 5-FU had no effect on intracellular oxidative stress or Ca^2+^ levels (Figure S3). These results suggest that quercetin may induce apoptosis in CRC cells by a 5-FU-independent pathway.

We next co-treated cells with quercetin and chemicals that regulate the physiology of intracellular Ca^2+^ to analyze changes in cell properties. Specifically, 2-aminoethoxydiphenyl borate (2-APB), which regulates the release of Ca^2+^ by inhibiting 1,4,5-trisphosphate (IP_3_) receptors in cells; ruthenium red (RR), which acts as an inhibitor of a mitochondrial Ca^2+^ uniporter; and BAPTA, which prevents intracellular Ca^2+^ overload, were co-treated with quercetin. Interestingly, in CRC cells, only RR restored the inhibitory effect of quercetin on cell viability to the control level (Figure 5E). We also found that RR attenuated the increase in both intracellular and mitochondrial ROS production caused by quercetin in HCT116 cells (Figure 5F,G). RR significantly mitigated the apoptosis rate that was increased by quercetin and attenuated the decrease in MMP caused by quercetin in HCT116 cells (Figure 5H,I). Furthermore, RR decreased the proportion of cells in the sub-G1 phase, which was increased by quercetin (Figure 5J). Therefore, we investigated the effect of RR on the expression of CCND1 and PCNA, proteins essential for cell cycle progression and proliferation (Figure 5K). RR co-treated with quercetin significantly upregulated the expression levels of CCND1 and PCNA; in addition, compared with treatment with quercetin alone, the expression levels of CTNNB1 were increased in HCT116 cells following co-treatment with RR. These results suggest that quercetin induces oxidative stress and targets mitochondria, leading to the induction of apoptosis and inhibition of cell growth, although further studies are needed to elucidate specific mechanisms in different cell types.

### 3.5. Quercetin Regulates the Expression Level of tiRNAs in CRC Cells and tiRNA^HisGTG^ Potentiates the Apoptotic Effect of Quercetin

**Figure 6:**
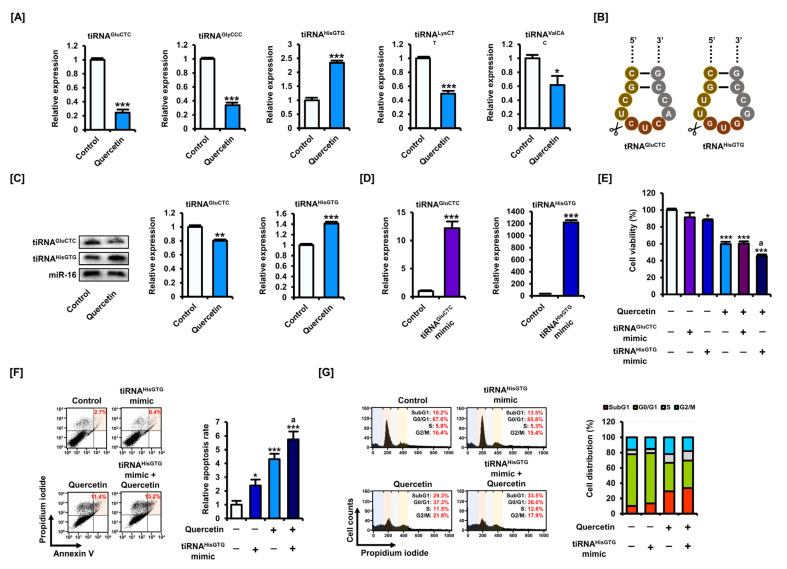
Quercetin regulates the expression of tiRNA in CRC cells. (**A**) Changes in candidate tiRNA expression following quercetin treatment in HCT116 cells were analyzed by qPCR. (**B**) Sequences and cleavage positions in the anticodon loops of tiRNA^GluCTC^ and tiRNA^HisGTG^. (**C**) Band images of tiRNA^GluCTC^ and tiRNA^HisGTG^ were detected using a DIG probe. Expression of miR-16 was used for normalization. (**D**) After transfection of mimics of tiRNA^GluCTC^ (10 nM) and tiRNA^HisGTG^ (10 nM) into HCT116 cells, increased expression was confirmed by qPCR. (**E**) Cell viability following transfection of mimics of tiRNA^GluCTC^ (10 nM) and tiRNA^HisGTG^ (10 nM) for 5 h and quercetin (40 μM) treatment was measured in HCT116 cells. (**F**) Apoptotic rates following tiRNA^HisGTG^ (10 nM) transfection for 5 h and quercetin (40 μM) treatment, as measured by dual Annexin V/propidium iodide (PI) staining and flow cytometry over 48 h. The percentage of cells corresponding to the upper right of the quadrant was used to compare the relative number of apoptotic cells. (**G**) Cell cycle distribution following tiRNA^HisGTG^ (10 nM) transfection for 5 h and quercetin (40 μM) treatment determined by PI staining and expressed as proportions of cells in sub-G1, G1, S, and G2/M phases over 48 h. Data are presented as representatives of the results of three independent experiments. Asterisks indicate statistically significant differences compared with untreated controls (*** *p* < 0.001; ** *p* < 0.01; * *p* < 0.05). The symbol ‘a’ indicates a significant difference between combination treatment and quercetin treatment alone (*p* < 0.05).

Based on previous reports, we selected five candidate tiRNAs presumed to show different expression levels in CRC than in normal tissues. We found that quercetin inhibited the expression levels of tiRNA^GluCTC^, tiRNA^GlyCCC^, tiRNA^LysCTT^, and tiRNA^ValCAC^ in HCT116 cells, but increased the expression levels of tiRNA^HisGTG^ (Figure 6A). We therefore determined the function of tiRNA^GluCTC^, which showed the greatest decrease in expression from quercetin, and tiRNA^HisGTG^, which showed increased expression in HCT116 cells (Figure 6D). Through small RNA band detection using a DIG probe, we reconfirmed that quercetin reduced the expression levels of tiRNA^GluCTC^ and upregulated the expression levels of tiRNA^HisGTG^ in HCT116 cells (Figure 6C). After transfecting mimics of tiRNA^GluCTC^ and tiRNA^HisGTG^ into HCT116 cells, high expression levels of each tiRNA was confirmed (Figure 6D). Cell viability assays suggested that the tiRNA^HisGTG^ mimic further enhanced the inhibition of cell viability caused by quercetin treatment in HCT116 cells (Figure 6E). However, the tiRNA^GluCTC^ mimic did not significantly alter the viability of HCT116 cells. Therefore, we speculate that tiRNA^HisGTG^ could further sensitize the effect of quercetin in a cell line-dependent manner. The transfection of the tiRNA^HisGTG^ mimic into HCT116 cells further enhanced the increased proportion of apoptotic cells caused by quercetin (Figure 6F). In addition, cell cycle analysis suggested that tiRNA^HisGTG^ contributed to the anticancer effect of quercetin by increasing the proportion of HCT116 cells in the sub-G1 phase (Figure 6G). These results suggest that the regulation of specific tiRNAs may contribute to the apoptosis-inducing effect of quercetin in CRC cells. Further research is needed to determine the detailed mechanisms underlying tiRNA generation and to clarify the role of tiRNAs in response to quercetin in CRC cells.

### 3.6. Oral Administration of Quercetin Induces an Inhibitory Effect on Tumor Growth in Colitis-Associated CRC Mice

**Figure 7:**
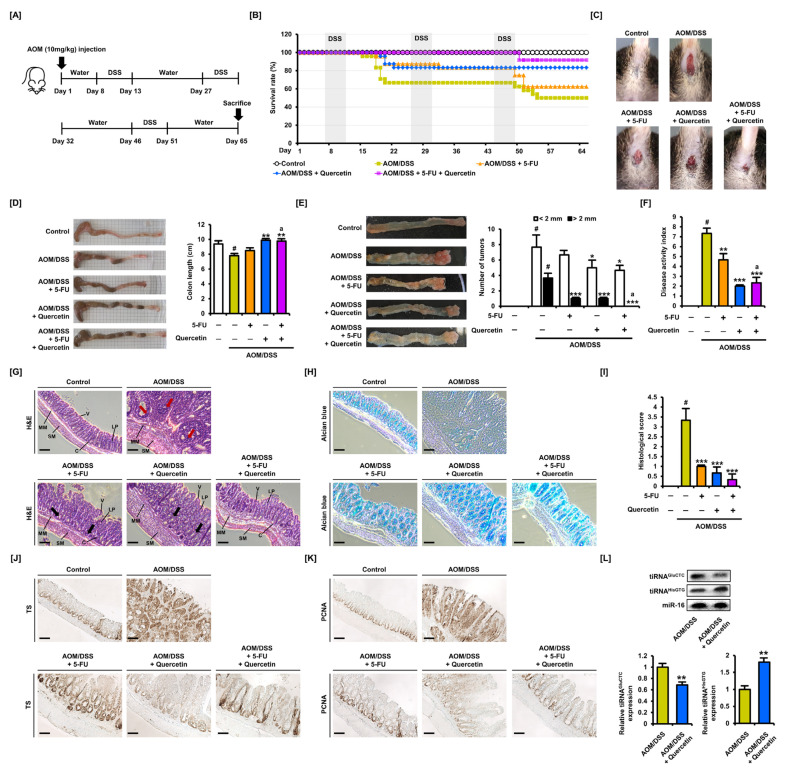
The inhibitory effect of quercetin on tumor growth was investigated in the colitis-associated CRC mouse model. (**A**) Schematic diagram of colitis-associated CRC mouse model established through AOM injection and oral supply of DSS. (**B**–**D**) Changes in survival rate (**B**), rectal bleeding (**C**), and colon length (**D**), according to administration of 5-FU and quercetin in CRC mice. (**E**) The number of tumors was counted according to the size of the tumor, based on the length of the long axis. (**F**) The disease activity index of CRC mice was quantified based on body weight loss, rectal bleeding, and stool consistency. (**G**) Hematoxylin and eosin (H&E) staining was performed to stain the nucleus and cytoplasm of distal colon tissues of CRC mice. V, villi; C, intestinal crypts; LP, lamina propria; MM, muscularis mucosae; SM, submucosa. Red arrows indicate tumor foci and black arrows indicate hyperplasia. (**H**) Alcian blue staining was performed to visualize goblet cells on distal colon tissues of CRC mice. (**I**) Histological scoring based on damage to crypts and goblet cells showed the effect of 5-FU and quercetin on intestinal damage. Histological scoring based on damage to crypts and goblet cells was performed to show the effect of 5-FU and quercetin on intestinal damage. (**J**) Immunohistological analysis was performed to visualize TS expression levels and localization in distal colon tissues of CRC mice. (**K**) Immunohistological analysis was performed to visualize PCNA expression level and localization in distal colon tissues of CRC mice. (**L**) Band images of tiRNA^GluCTC^ and tiRNA^HisGTG^ were detected using a DIG probe in distal colon tissues of CRC mice. Expression of miR-16 was used to normalize data. Data are presented as representatives of the results of three independent experiments. Scale bar indicates 100 μm. Asterisk indicates a statistically significant difference compared with untreated AOM/DSS controls (*** *p* < 0.001; ** *p* < 0.01; * *p* < 0.05). The symbol ‘#’ indicates a significant difference between normal and AOM/DSS controls (*p* < 0.05). The symbol ‘a’ indicates a significant difference between combination treatment and 5-FU alone (*p* < 0.05).

To investigate the effect of quercetin on CRC in vivo, we established a colitis-associated CRC mouse model by intraperitoneally injecting AOM and delivering DSS in drinking water (Figure 7A). The survival rate of CRC mice not treated with 5-FU or quercetin was 50% over 65 days (Figure 7B). The intraperitoneal injection of 5-FU increased the survival rate to 62.5% and oral administration of quercetin increased the rate to 83.3%. Moreover, the combined treatment of 5-FU and quercetin increased the survival rate of CRC mice to 91.7%. We found that a large number of CRC mice died 7 or 8 days after the initial DSS treatment, whereas few died following the second DSS cycle. A large number of CRC mice also died immediately after the third DSS cycle, especially in the group injected with 5-FU. These results suggest that 5-FU may be less effective in protecting mice from advanced colorectal cancer. We also found that neither 5-FU nor quercetin influenced weight loss in CRC mice (Figure S4A). We observed rectal bleeding as evidence of colonic injury in CRC mice (Figure 7C) and found that 5-FU or quercetin alone relieved rectal bleeding and combination treatment significantly restored colon damage. In addition, quercetin relieved the symptoms of bloody stool and diarrhea in CRC mice (Figure S4B). Treatment with 5-FU did not significantly improve the decreased colon length of CRC mice, but the oral administration of quercetin restored colon length to the control level (Figure 7D). Moreover, by classifying tumors according to size, we found that 5-FU and quercetin inhibited the growth of tumors larger than 2 mm (Figure 7E). Tumorigenesis was especially concentrated in the distal colon. Next, we presented the DAI by scoring the symptoms of colon damage based on weight loss, rectal bleeding, and stool consistency (Figure 7F). Compared with 5-FU alone, the addition of quercetin significantly alleviated DAI in CRC mice.

We collected the distal colon and performed histological analysis. H&E staining showed that tumors formed in the AOM/DSS CRC model mice and that their colons were thickened (Figure 7G). The administration of 5-FU inhibited tumor formation, but a wide range of mucosal damage was observed. The administration of quercetin also inhibited tumor formation, but compared with that of the control group, the thickness of the colon was greater. We confirmed that intestinal goblet cells in the tissues of CRC mice were damaged by staining the tissues with Alcian blue, which detects acidic mucin (Figure 7H). The administration of 5-FU and quercetin increased the proportion of normal goblet cells. Histological damage was scored based on the extent of the damage to the mucosa and goblet cells in colon tissues (Figure 7I). We found that 5-FU and quercetin significantly alleviated colonic damage caused by AOM/DSS in mice. Next, we performed immunohistochemical analysis to determine the expression levels and localization of TS in the colon tissues of CRC mice (Figure 7J). High expression levels of TS were observed in tumors from mice administered with AOM/DSS. Mice administered with 5-FU maintained high expression levels of TS, whereas quercetin suppressed the expression of TS. In addition, the expression levels of PCNA, a marker of cell proliferation, were high in CRC mice, and this expression was suppressed by quercetin (Figure 7K). Finally, we analyzed the expression levels of tiRNA^GluCTC^ and tiRNA^HisGTG^ following quercetin administration in CRC mice (Figure 7L). We found that expression levels of tiRNA^GluCTC^ were suppressed, and expression levels of tiRNA^HisGTG^ were increased, consistent with the results found in CRC cells. These results suggested that quercetin may serve as a therapeutic adjuvant to 5-FU by targeting TS to inhibit tumorigenesis, although the in vivo regulatory function of candidate tiRNAs on tumor growth requires further study.

### 3.7. Quercetin Alleviates the Inflammatory Response Induced by 5-FU in Colitis-Associated CRC Mice

**Figure 8:**
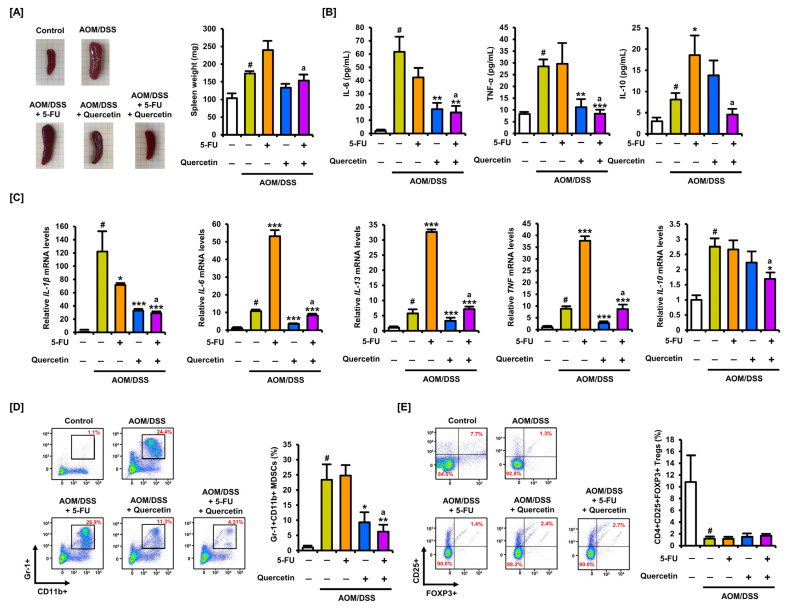
Quercetin ameliorated 5-FU-induced increases in inflammatory cytokines and increases in myeloid-derived suppressor cells (MDSCs) in CRC mice. (**A**) Spleen images following administration of 5-FU and quercetin in CRC mice are presented. (**B**) Concentration changes in IL-6, TNF-α, and IL-10 following administration of 5-FU and quercetin in the serum of CRC mice were analyzed by ELISA. (**C**) Changes in mRNA expression levels of IL-1β, IL-6, IL-13, TNF, and IL-10 following administration of 5-FU and quercetin in distal colon of CRC mice were analyzed by qPCR. (**D**) The proportion of MDSCs positive for Gr-1 and CD11b in the spleen of CRC mice was determined by flow cytometry. (**E**) The proportion of Tregs positive for CD4, CD25, and FOXP3 in the spleen of CRC mice was determined by flow cytometry. Data are presented as representatives of the results of three independent experiments. Asterisks indicate statistically significant differences compared with untreated AOM/DSS controls (*** *p* < 0.001; ** *p* < 0.01; * *p* < 0.05). The symbol ‘#’ indicates a significant difference between normal and AOM/DSS controls (*p* < 0.05). The symbol ‘a’ indicates a significant difference between combination treatment and 5-FU treatment alone (*p* < 0.05).

As 5-FU administration can induce an inflammatory response in the colon, we investigated whether quercetin could inhibit the induction of inflammation by 5-FU. First, we confirmed that the size of the mouse spleen was increased by AOM/DSS administration (Figure 8A). Treatment with 5-FU did not affect the weight of the spleen in AOM/DSS CRC mice, but spleen weight was reduced by co-treatment with quercetin. Next, we analyzed the amounts of inflammatory cytokines in CRC mouse serum using ELISA (Figure 8B). As expected, the amounts of IL-6, TNF-α, and IL-10 were increased in AOM/DSS CRC mice compared with controls. Although 5-FU did not significantly affect the expression levels of these cytokines, their amounts were significantly reduced under combination treatment with quercetin. Moreover, we measured the mRNA levels of several cytokines using quantitative PCR (Figure 8C). We found that the administration of 5-FU significantly increased the mRNA levels of *IL-6*, *IL-13*, and *TNF* in CRC mice. Compared with 5-FU alone, the combined treatment with quercetin reduced the mRNA levels of all measured cytokines. These results suggest that quercetin can alleviate the inflammatory response caused by 5-FU during CRC treatment. Next, we analyzed changes in the ratio of MDSCs and Tregs in the spleen of CRC mice following administration of 5-FU and quercetin. We found that the amount of MDSCs dramatically increased in AOM/DSS CRC mice compared with that of the control group (Figure 8D). The proportion of MDSCs was not affected by 1-FU, but quercetin alone or in combination with 5-FU attenuated the increase in MDSCs. The ratio of Tregs was significantly decreased in AOM/DSS mice compared with that of controls, but there was no significant change in response to treatment with 5-FU and quercetin (Figure 8E). These results suggest that quercetin may modulate the proportion of specific immune cells involved in tumor growth.

## 4. Discussion

The most common chemotherapeutic agent used for the treatment of CRC is 5-FU, and it functions by irreversibly inhibiting TS, causing DNA damage [23]. It is well-known that inhibition of TS activity causes antiproliferative effects accompanied by cell cycle arrest [24]. A previous study revealed that quercetin could inhibit TS, regulate the cell cycle, and induce apoptosis in oral cancer cells [25]. As previously reported, 5-FU increases the expression levels of both bound and unbound TS in CRC cells, presumably in a feedback loop to protect cells from drugs that inhibit TS activity [26]. In this study, quercetin inhibited the expression of TS in CRC cells and enhanced their sensitivity to 5-FU. The changes in TS expression levels in response to 5-FU and quercetin were validated in the tissues of CRC mice. In addition, the effects of 5-FU resistance on cell viability and cell proliferation were attenuated by the combined treatment of quercetin with 5-FU, as demonstrated in HCT116-5-FUR cells. However, the apoptosis-inducing effect of quercetin differed between cells harboring wild-type p53 and those harboring mutant p53. As p53 is a key protein involved in apoptosis induction and cell cycle arrest, the effect of quercetin is thought to be insignificant in CRC cells harboring mutant p53.

p53-mediated regulation of the p21 promoter is an important therapeutic target in CRC [27]. In CRC cells harboring mutant p53, such as HT29 cells, transcription of p21 is inhibited and p21-induced cell cycle arrest is avoided. A previous study provided evidence of a potential synergistic effect of 5-FU with quercetin and its dependence on the p53 status in CRC cells [7]. Moreover, treatment with 5-FU and quercetin alone or in combination increased expression of p53 and induced cleavage of caspase-9, caspase-3, and PARP in CRC cells [7,28]. Meanwhile, in CRC cells exposed to 5-FU, Ca^2+^ acts as a messenger for p53 activation [29]. The increase in quercetin-induced apoptosis and decrease in CCND1 and PCNA expression levels were restored to control levels by an inhibitor of the Ca^2+^ mitochondrial uniporter, suggesting that mitochondrial Ca^2+^ may be involved in quercetin-induced apoptosis. As the mechanism of quercetin-induced oxidative stress and Ca^2+^ dysregulation is not regulated by 5-FU, it is notable that quercetin causes apoptosis in 5-FU-resistant CRC cells. The induction of oxidative stress, representative of ROS generation, is a promising therapeutic strategy to promote cancer cell death. Cancer cells can be specifically targeted by taking advantage of the fact that ROS levels remain high in cancer cells, compared with that of normal cells, and inducing excessive intracellular ROS [30,31,32,33]. Quercetin was found to induce high levels of intracellular and mitochondrial-specific ROS in CRC cells, and this increase was independent of the status of p53. These results highlight the value of quercetin as a potential therapeutic adjuvant, as it can effectively induce oxidative stress in CRC cells.

We found that the tumor inhibitory effect and regulatory mechanisms of quercetin that were verified in CRC cell lines were also reflected in colitis-associated CRC mice. It is well-known that quercetin inhibits the progression of inflammatory diseases by inhibiting adverse inflammatory signals. However, it was unclear whether excessive induction of the inflammatory response by 5-FU treatment could be inhibited by quercetin in the inflammatory CRC mouse model. In this study, quercetin decreased the serum and mRNA levels of inflammatory cytokines that were increased by 5-FU in CRC mice. However, in the CRC mice used in this study, quercetin did not significantly modulate the anti-inflammatory mediator IL-10 at the mRNA and protein levels, thereby necessitating further sophisticated analysis. Meanwhile, recent evidence has suggested that MDSCs can promote tumor growth in the tumor microenvironment, making them a useful therapeutic target [34]. We found that quercetin administration alleviated the increase in MDSCs in the spleens of colitis-associated CRC mice. These results suggest that quercetin may induce a tumor-suppressive effect by modulating the composition of immune cells in the tumor microenvironment, although quercetin did not affect the proportion of Tregs.

Administration of 5-FU inhibits DNA and RNA synthesis and regulates the expression of non-coding RNAs (ncRNAs), including miRNAs, in CRC cells. ncRNAs either promote or alleviate 5-FU resistance depending on their target genes [35]. Therefore, modulation of ncRNAs by therapeutic agents is considered a strategy for overcoming 5-FU resistance in CRC cells. MicroRNAs (miRNAs) are the most widely known functional ncRNAs in cells that target TS in CRC and affect the therapeutic efficacy of 5-FU-based chemotherapy [36]. For example, miR-375-3p directly modulates TS in CRC cells, resulting in an effect similar to TS knockdown [37]. MiR-197, which is negatively correlated with TS expression in patients with advanced CRC, can also target TS and ameliorate TS-induced 5-FU resistance in CRC cells [38]. Meanwhile, under stress conditions, angiogenin translocates to the cytoplasm and promotes cell survival by specifically cleaving the anticodon loop of tRNA, leading to the inhibition of translation initiation and activation of a stress protective response [39]. The physiological effects of tiRNAs produced by the cleavage of half of the tRNA have recently been reported in various cell types [40,41]. Although there have been no reports that tiRNAs directly regulate TS, recent studies suggest that tiRNAs may be therapeutic targets in CRC cells. A specific tiRNA generated by the HIF1α/ANG axis in response to hypoxia may target *LATS2* to regulate CRC progression [42]. Several large-scale small RNA sequencing analyses have provided a list of tiRNAs with significantly altered expression in CRC cancer patient tissues [43,44]. In particular, bioinformatics analysis has shown that tiRNA^ProTGG^ levels are clinically associated with poor prognosis and recurrence in patients with CRC [12]. One sequencing study of CRC tissues suggested that, of the candidate tiRNAs analyzed in this study, only tiRNA^HisGTG^ was expressed at lower levels in tumor tissues than in adjacent tissues [11]. The results of this study and those of a large-scale RNA analysis suggest that tiRNA^HisGTG^, unlike the majority of other tiRNAs, may be involved in tumor growth inhibition. In our study, tiRNA^HisGTG^ decreased the viability of CRC cells and caused an increase in apoptosis. In contrast, the expression of tiRNA^GluCTC^ was significantly reduced by quercetin; however, tiRNA^GluCTC^ itself did not affect the viability of CRC cells. Therefore, we propose that among several tiRNAs, a specific tiRNA derived from tRNA^HisGTG^ is a target ncRNA associated with the efficacy of quercetin and may have anticancer effects. A significant increase in tiRNA^HisGTG^ expression by quercetin in colitis-associated CRC mice complements this hypothesis. Research on the function of specific tiRNAs in cancers including CRC is still in its early stages; therefore, more detailed research is needed in the future. Further studies are needed to elucidate whether specific tiRNAs, including tiRNA^HisGTG^, can directly target TS in CRC cells.

For future clinical use of quercetin as an adjuvant, the appropriate concentrations to be applied to the patient should be considered. In clinical trials of quercetin in various diseases, approximately 1000 mg/day of quercetin was metabolized in the body without any reported side effects [45,46,47]. Therefore, the concentrations and frequencies of application that cause no side effects in the clinical application of quercetin are well-established. However, treatment efficiency and resistance improvement should be verified when used with standard anticancer drugs, including 5-FU, in such applications.

## 5. Conclusions

This study investigated the effects and mechanisms of quercetin in enhancing the sensitivity of CRC cells to 5-FU. Although this study investigated the inhibitory effects of quercetin on tumor growth in colitis-associated CRC mice, the findings were limited in that the effect of tiRNAs on tumor progression was not revealed in vivo. In addition, further studies are needed to elucidate the effect of quercetin-regulated MDSCs on tumor progression. Overall, this study demonstrated a novel use for quercetin in enhancing the effects of 5-FU in CRC, and identified various physiological and molecular changes caused by quercetin in the tumor and tumor microenvironment.

## Data Availability

The data are contained within the article and Supplementary Materials.

## Supplementary Materials

The following supporting information can be downloaded at: https://www.mdpi.com/article/10.3390/antiox11112158/s1, Figure S1: Dose-dependent effects of 5-FU (0, 1, 2, 4, 8, 16, 32, and 64 μM) for 48 h on the viability of HCT116-5-FUR cells; Figure S2: Dose-dependent effects of quercetin (0, 10, 20, and 40 μM) for 1 h on superoxide production in HCT116 and HT29 cells; Figure S3: Effects of 5-FU and quercetin on ROS production and intracellular Ca^2+^ levels; Figure S4: Effects of 5-FU and quercetin on body weight and stool consistency in the colitis-associated CRC mouse model.
