# Supplementary material for: Targeting Thymidylate Synthase and tRNA-Derived Non-Coding RNAs Improves Therapeutic Sensitivity in Colorectal Cancer
Source: Antioxidants (Basel). 2022 Oct 31;11(11):2158. doi: 10.3390/antiox11112158 (PMC9686910; doi:10.3390/antiox11112158)
Supplement: Supplementary file 1 [file antioxidants-11-02158-s001.zip › antioxidants-1955938-supplementary.pdf]

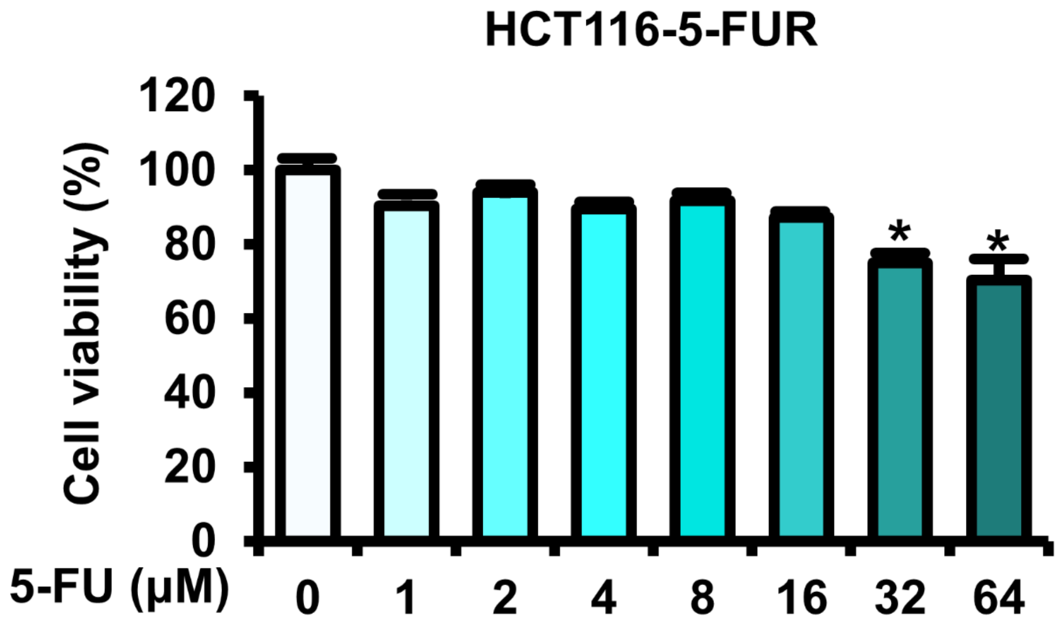

Fig. S1. Dose-dependent effects of 5-FU (0, 1, 2, 4, 8, 16, 32, and 64 μM) for 48 h on the viability of HCT116-5-FUR cells. Asterisks indicate statistically significant differences compared to untreated controls ($*p < 0.05$).

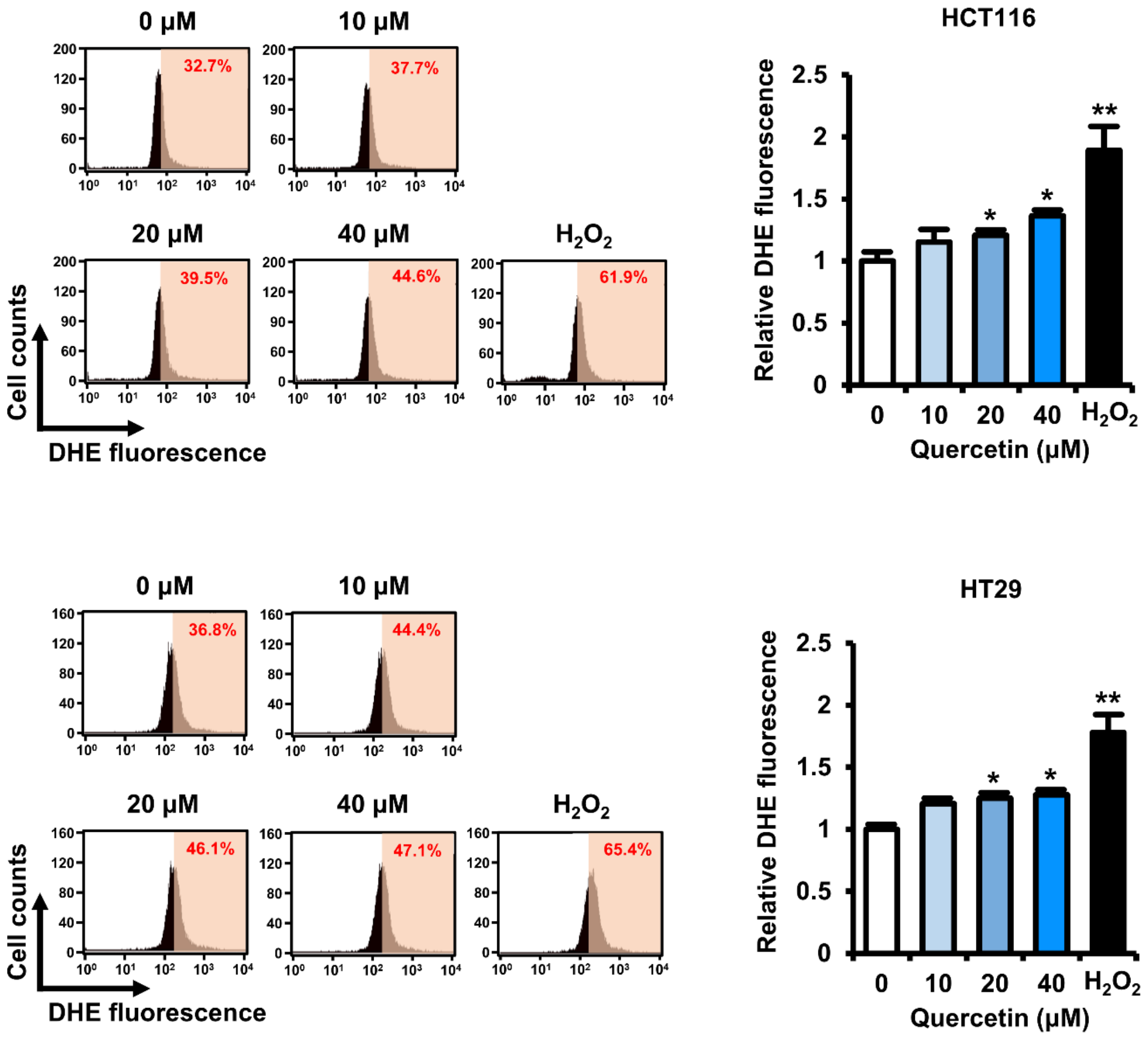

Fig. S2. Dose-dependent effects of quercetin (0, 10, 20, and 40 μM) for 1 h on superoxide production in HCT116 and HT29 cells. DHE fluorescence values were estimated by flow cytometry. Asterisks indicate statistically significant differences compared to untreated controls (**$p < 0.01$; *$p < 0.05$).

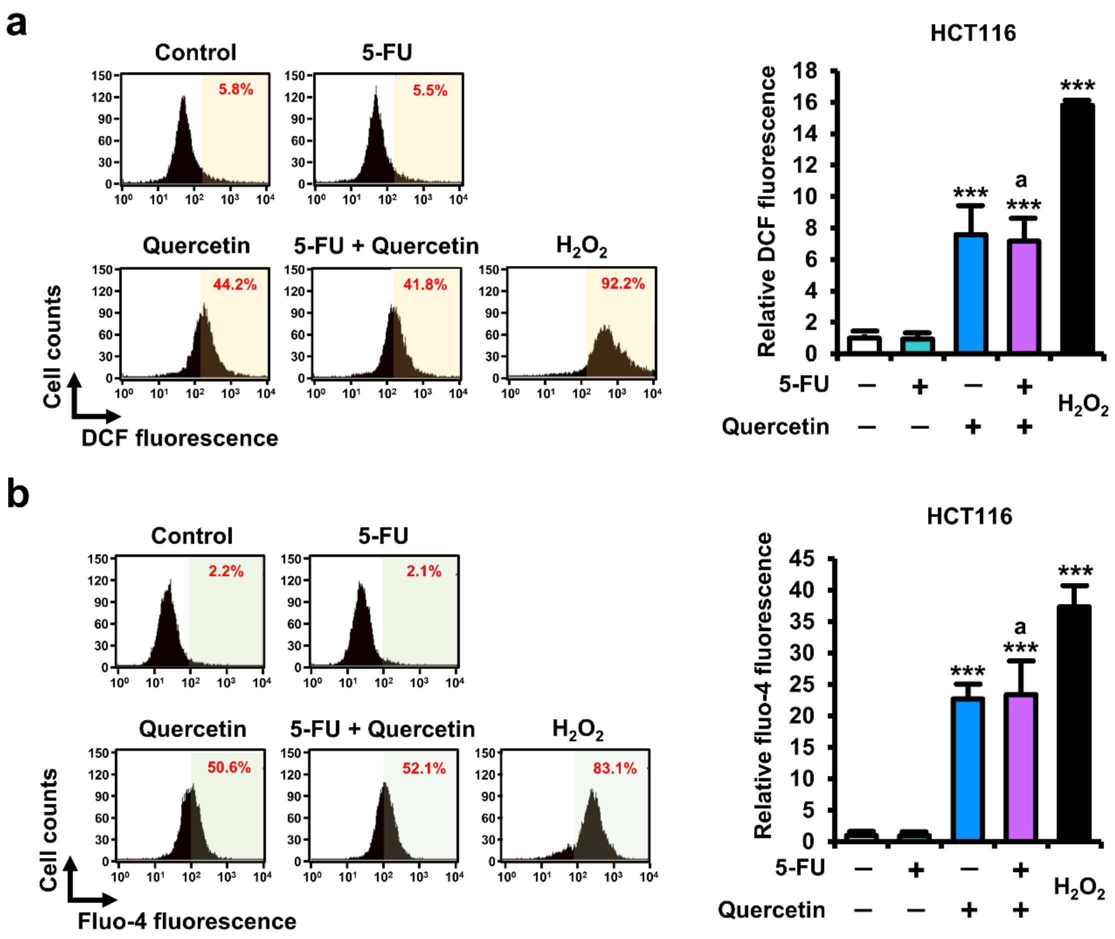

Fig. S3. Effects of 5-FU and quercetin on ROS production and intracellular Ca^{2+} levels. (a) Effects of 5-FU (20 μM) and quercetin (40 μM) for 1 h on ROS production in HCT116 cells. DCF fluorescence values were estimated by flow cytometry. (b) Effects of 5-FU (20 μM) and quercetin (40 μM) for 1 h on intracellular Ca^{2+} levels in HCT116 cells. Fluo-4 fluorescence values were estimated by flow cytometry. Asterisks indicate statistically significant differences compared to untreated controls (***$p < 0.001$). The symbol 'a' indicates a significant difference between combination treatment and 5-FU treatment alone ($p < 0.05$).

a

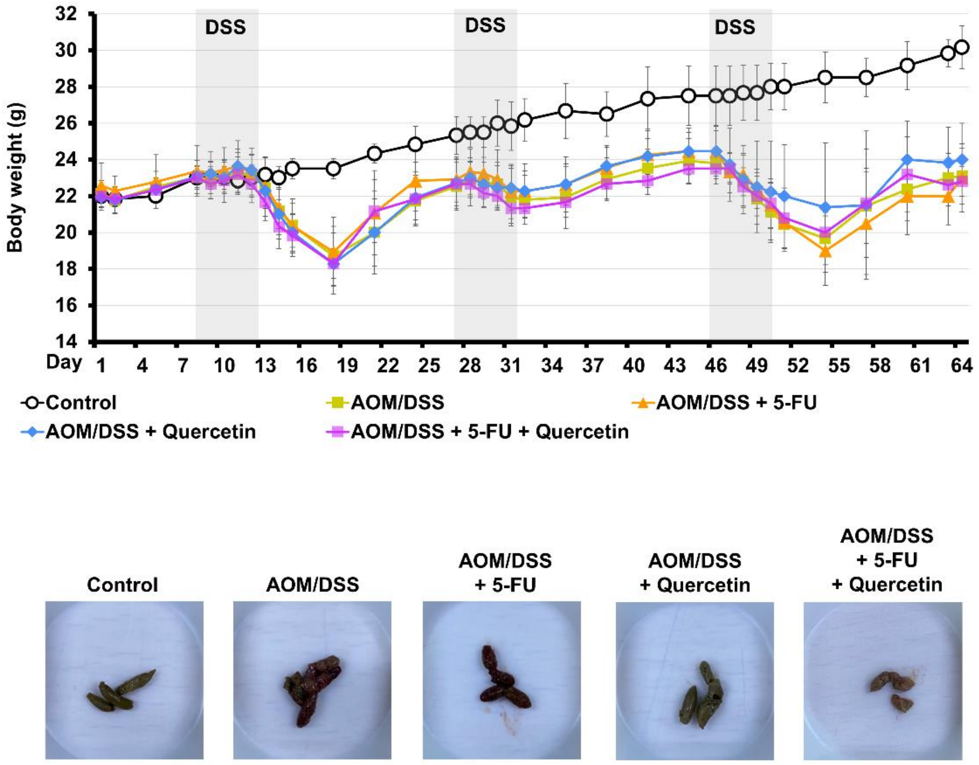

Fig. S4. Effects of 5-FU and quercetin on body weight and stool consistency in the colitis-associated CRC mice model. (a) Changes in body weight according to administration of 5-FU and quercetin in CRC mice. (b) Changes in stool consistency according to administration of 5-FU and quercetin in CRC mice.
